# Challenges and strategies for recruitment of minorities to clinical research and trials

**DOI:** 10.1017/cts.2023.559

**Published:** 2023-06-13

**Authors:** Susan P. Fisher-Hoch, Jennifer E. Below, Kari E. North, Joseph B. McCormick

**Affiliations:** 1 UTHealth Houston School of Public Health, Brownsville Campus, Brownsville, TX, USA; 2 Vanderbilt University Medical Center, Division of Genetic Medicine, Nashville, TN, USA; 3 University of North Carolina Gillings School of Global Public Health, Chapel Hill, NC, USA

**Keywords:** Recruitment, Chronic disease, minority clinical studies, community engagement, health disparities

## Abstract

Minority populations are largely absent from clinical research trials. The neglect of these populations has become increasingly apparent, with escalating cancer burdens and chronic disease. The challenges to recruitment of minorities in the United States are multiple including trust or lack thereof. Keys to successful recruitment are responding to community issues, its history, beliefs, and its social and economic pressures. The strategy we have used in many low-income, sometimes remote, communities is to recruit staff from the same community and train them in the required basic research methods. They are the first line of communication. After our arrival in the Texas Rio Grande Valley in 2001, we applied these principles learned over years of global research, to studies of chronic diseases. Beginning in 2004, we recruited and trained a team of local women who enrolled in a cohort of over five thousand Mexican Americans from randomly selected households. This cohort is being followed, and the team has remained, acquiring not only advanced skills (ultrasound, FibroScan, retinal photos, measures of cognition, etc.) but capacity to derive key health information. Currently, we are participating in multiple funded studies, including an NIH clinical trial, liver disease, obesity, and diabetes using multiomics aimed at developing precision medicine approaches to chronic disease prevention and treatment.

## Introduction

It has long been recognized that inclusion of minority populations in clinical trials has been lacking [1]. The FDA has recognized the need for broader inclusion over the years, but until recently, their list emphasized mainly age, sex, and particular conditions. In 2020, new recommendations proposed broadening inclusion criteria, modifying trial design to better accommodate diverse populations, and decreasing patient burdens, but this has been slow going. These problems appear particularly true for therapeutic trials within existing sites entrenched in places where recruiting minorities may be practically difficult, with strict inclusion criteria limiting participation in poorer communities. The neglect of these populations has become increasingly apparent, with cancer burdens and COVID-19 incidence and mortality escalating in minority populations, in comparison to their white counterparts. For example, black populations have more than double the mortality of non-Hispanic white populations from prostate cancer, and similarly, Hispanics have more than double the mortality from liver cancer, when compared to non-Hispanic white populations [2], the reasons for which are little understood largely due to lack of focused studies. Chronic conditions which predispose to cancer and other potentially fatal complications, such as obesity and metabolic diseases, have elevated prevalence in some minority communities [3,4]. Nevertheless, in 2020, 73% of subjects in U.S. clinical trials leading to new drugs were listed as white, 5% as black, and 14% as Asian. Only 6% were Hispanics, despite their comprising nearly 20% of the population [1,5].

Chronic diseases such as hypertension, renal failure, and type 2 diabetes also disproportionately affect minorities [6]. The genetic, biological, and sociodemographic elements driving these higher rates are poorly understood and it is becoming increasingly clear that the causes are highly dynamic and may even differ by genetic background. Thus, there is great need for targeted clinical trials focused on the populations most at risk in whom the etiology and processes may differ from those in whites, necessitating different preventive and therapeutic approaches.

Many important studies provide excellent examples of successful research in distinct communities, for example, vaccine studies in low-income populations. An important recent example of a successful clinical trial is that of a malaria vaccine in Ghana, Kenya, and Malawi, where more than a million children were vaccinated [7]. Such studies conducted by pharma in collaboration with WHO and other major agencies have been successful for both dengue and HIV in Africa and elsewhere. The success of these studies may reflect the high prevalence of disease in these populations and therefore the community’s motivation to find successful preventions and treatments. Most importantly, these trials worked closely with local researchers who understand their communities and can gain access, often using community outreach workers.

### Challenges in communities

The challenges to recruitment of minorities in the United States are multiple and riddled with complexities. Though many of the challenges have common origins, their manifestations differ between communities and in space and time. The most important element is trust or lack thereof. Thus, the key to successful recruitment is understanding the issues of the community, its history, its beliefs, and the social and economic pressures to gain that trust. Distrust among the African American community was originally fueled by highly visible transgressions, for example, the Tuskegee syphilis study [8,9]. Though this notorious study was conducted nearly a century ago, this and other cases of abuse have led to long-term distrust of the medical community that persists. Among Hispanics, distrust is fueled by documentation and deportation fears. Distrust is compounded in many places by poverty. Hourly wage earners cannot spare time to attend a clinic leading to poor participation, particularly by men. Women with extensive household responsibilities may not have time to participate. These communities also often lack access to quality health care. Studies that require Internet access for the subject present another major problem frequently not appreciated in centralized large studies, since the lowest income individuals often rely on smartphones for all communications and would not trust a website or use or even own a computer. People in disadvantaged communities change phone numbers and addresses frequently and thus may be difficult to follow-up in a trial. There are language barriers, environmental issues, racism, and social and economic discrimination. Environmental issues such as housing, security, and transportation as well as other social issues are significant barriers. There are also multiple distractions in the quest for daily survival. Often the disadvantaged have had limited access to education such that the importance of science behind health decisions may be difficult for them to grasp, leading to reliance on misinformation or misguided opinions of family, friends, media, or entrenched customs and beliefs. These multiple challenges need to be understood before designing a trial to enhance success.

### Challenges presented by investigators

Failure to appreciate and confront these barriers is a major reason for failure to recruit. Added to this are other factors under the control of the investigators. First, lack of communication, particularly listening to the community in the design process. Lack of feedback to the community and little continued communication will also discourage participation. Any attempt to run a complex study from a distance is also doomed to be a failure. Onsite senior investigators need to be constantly available to navigate problems related to recruitment success and protocol adherence. Failure to understand these needs may be ignorance; sometimes arrogance will be compounded by poor communication. This has at times been termed “parachute science;” a negative term for scientific studies in minority communities run from afar, designed to serve the scientist and not the community.

### Strategies for success

Working with community members at the design stage cannot be overemphasized. If the project does not meet any community needs even if only in terms of future health, it may be difficult to garner enthusiasm. Couching the goals of the project in terms the community members can understand is key. Working with the community is a two-way process. What the community perceives as a problem may well not be what the investigator proposes to study. This requires dialogue so that each understands the other. The strategy we have used in many low-income, sometimes remote, communities has been to recruit staff from the community and train them in the basic research methods required for the study. Let these community staff persons be the first line of communication. For this purpose, even individuals with limited education, essentially outreach workers, can be taught how to perform the tasks and explain issues, and with support, they are able to do these very well. They are then better equipped to go into the community and recruit subjects since they are much more easily acceptable locally and speak the same language and share the same culture, which the investigators often do not. It is important to get the input of these staff into language of informed consent so that subjects can understand the questions. Incentives are important but often are better appreciated in forms that the subjects need, such as free laboratory results, gift cards, rather than cash. An important issue is to spend time discussing the projects with the subjects as they are recruited and returning to them data gained in a user-friendly format. Communicating results to the community at large is also important. Lastly, the team of local staff needs to feel some ownership in the project and appreciate that they are serving the community as well as science. The walls of the Clinical Research Unit are lined with posters illustrating the results of the work the staff performs on a daily basis, involving them and giving them ownership in this and other ways. We discuss results with our staff to ensure they understand the importance and results of their work.

Procedures need to be designed so that there is minimal burden on the research subjects. In communities with limited access to Internet, personal approaches, telephone, and even house calls are important in gaining trust and enhancing participation. A powerful tool being used increasingly frequently is telemedicine. This also needs to be kept simple, and the smartphone is becoming ubiquitous and the means of communication of choice for minority communities. Telemedicine approaches can also be effectively used by outreach workers directly to the subject’s home requiring much less time for the subject

### Experiences and stories

The authors of this paper have learned these lessons over many years of working in remote areas of Africa, rural China, and urban South Asia. Many issues are immediately translatable to the continental US, and many are not, but they give us a good starting point. An early study in Sierra Leone resulted in some unexpected reactions: local people thought if we took blood, we must be selling it for profit. This we had to overcome. We recruited local people with language skills since the tribal system required many people to speak their native tribal language and a lingua franca, often a version of pidgin English. In Sierra Lone, Krio is the lingua franca, but the people we recruited spoke Mende. Routine processes, such as informed consent, could only be verbal in a population where many could not read or write, and it had to go through at least two languages, sometimes three. For this reason, the terms and expressions had to be very basic and use language the subject understood. For instance, feeling sick in Krio is best expressed as “De body no good-o,” and the language has neither past nor future tenses. Getting a US-based IRB to fathom this was a major obstacle. In Sierra Leone, there was no IRB at the time, and we had to work with the Ministry of Health and local officials to introduce the concept and then create and train an IRB. In other locations, we have illustrations of how an effective approach can overcome barriers, for example, we have obtained data on depression, abuse, and sexual practices, and information on needle use from both patients and providers in urban and rural Pakistan.

People recruited locally as research workers, usually with limited formal education, but if their contribution is treated with respect, can achieve very high standards even with relatively complex tasks. Communities are disadvantaged, not ignorant, which is a good mantra to remember whenever working with minorities. Using this approach we were able to conduct a range of groundbreaking studies of the epidemiology and clinical disease of Lassa fever, in an area with little or no electricity or communications [10,11]. Subsequently, we were able to conduct and complete a clinical trial of ribavirin and compare it to the less successful immune plasma infusion for the first successful treatment of a viral hemorrhagic fever against the background described above [12]. Clearly, the nature of the disease and its severity also play a role in participation as is the case for Lassa fever in West Africa.

Further experience studying hemorrhagic fever with renal syndrome (HFRS: caused by a Hantavirus) in rural China, used the same approach, working through local health workers, familiar with the community. We took blood samples in these studies, despite the belief of rural Chinese, that they needed all their blood and could not spare any. A successful study of ribavirin treatment of HFRS by a different group in China was also successful [13]. Subsequently, this knowledge was important in managing and treating a related hantavirus infection, hantavirus pulmonary syndrome in the United States. Today, ribavirin is used widely for HFRS [14] in China and throughout China and the Middle East for a related hemorrhagic fever, Crimean, Congo Hemorrhagic Fever (CCHF). CCHF is now routinely treated in endemic regions from Turkey to China with ribavirin using an oral formulation from China [15]. We later conducted a study in downtown Karachi on the efficacy of parenteral polio vaccine (IPV) in neonates. This area was extremely poor and crowded with no street names. To follow-up babies born in city hospital and recruited there, our trained community health worker staff had to follow the mother home in order to conduct follow-up visits [16]. These examples of clinical trials conducted in remote areas with sufficient disease burden to derive efficacy data show the global value of such studies and the flexible culturally adapted approach needed to achieve good results.

After our arrival to South Texas Rio Grande Valley in 2001, we have applied these principles to studies of chronic diseases in the U.S./Mexico border. Beginning in 2004, we have recruited and trained a team of local women and they have been critical to enrolling a cohort of more than five thousand moderate to low-income Mexican Americans from randomly selected households. Our staff goes into the randomly selected blocks and tracks and systematically visits houses to engage families and explain the purpose of our work and to invite them to voluntarily participate. The staff also conducts all of the follow-up studies and the consistency of our staff interaction with our participants is key to comprehensive participation, retention, and follow-up. Our staff are bilingual in Spanish and English and look and talk like the participants they are recruiting. Personal recruitment is essential in this culture. Doors are opened for them, which would never be opened for the investigators [4,17]. This cohort has been followed since 2004, and the team has remained with the project, acquiring not only advanced skills (ultrasound, FibroScan, retinal photos, measures of cognition, mental health, DEXA) but are also able to communicate with people and deriv information that otherwise might be withheld [18–22]. Currently, we are conducting an NIH-funded study using a semaglutide in prediabetes, and recruitment in the hands of our staff is not a problem. Some participants often consider us a medical home, and since many do not have medical insurance of any kind, this is of great value to the participants and the investigators. The sense of community is strong across the whole project. We applied the same principles to recruitment of our laboratory and data management staff, which has further strengthened the community basis of our program.

## Discussion

The purpose of this brief report was to share the evolution of our experience in recruiting people who may otherwise be excluded from clinical research because of cultural, economic, linguistic, or geographic reasons. In any situation, the first rule for success is to know and understand the community that is the focus of the study and make sure they understand and are prepared to participate in the goals and understand the purpose of the science. We have found an effective way to recruit research workers from the community and train them well to communicate the purpose of the study to the participants and to learn to administer key questionnaires and other procedures in a culturally effective way. It is imperative to listen to and take the advice of the staff. We feel it is also very important that the staff feel ownership of the work they conduct. For that, we share the results of the work of the staff, which they can then share with the participants. Our CRU is lined with posters presented at meetings that show the results of their work. Partnership with community organizations from nonprofits to Federally Qualified Health Centers, to local officials and health departments in designing and running community research and outreach projects is very important and has been a hallmark of our participation in the CTSA program. Though not used for recruitment by us, an example is our Community Advisory Board, now run independently and renamed the Community Action Board where public health is discussed, and information and needs are exchanged. A growing component of our CTSA Center for Clinical and Translational Science (CCTS) is a “community scientist” program designed to bring researchers into direct contact with trained community members who can discuss and provide input on project design. The COVID-19 pandemic provided an extraordinary opportunity for our School of Public Health staff and faculty to work with local political, business, and health leaders to improve testing and vaccination in our community. This has resulted in an extremely high level of testing and vaccination against SARS-CoV2 virus in our community (∼98%).

Clinical studies conducted in culturally appropriate settings in disparate minority communities are becoming increasingly important and more common as health researchers are understanding that different contexts influence both disease manifestations and responses to therapies. Tailoring medications, interventions, and patient care to take account of these differences will be increasingly important as new discoveries reach the population. Precision medicine will need to take account of these influences. Some drugs will likely be developed that are specifically designed for individual genetic backgrounds. Finally, new advances in therapeutics and precision medicine should be available to all and the best route to achieve this is rigorous clinical trials in all types of communities.

A key issue to working with underserved minorities is to bring the same cutting-edge science that other more often included groups enjoy. One reason for this is to understand the unique issues that may apply to this population. For example, we find that average insulin resistance in our population is higher than in other populations. Further, about 25% of those with high levels of insulin resistance have no signs of other cardiometabolic risk factors, which usually cooccur with insulin resistance. We have the opportunity to explore these unique population-specific patterns of disease. Achieving comprehensive epidemiological studies from cell to society requires collaborations with individuals and institutions with expertise that we do not possess. Our now 20+ year program includes a range of cutting-edge longitudinal molecular characterizations to assess pathways associated with acquisition of early signs of disease. Thus, we are able to work with colleagues such as Drs. Below and North, coauthors of this paper, to pursue the understanding of pathways of disease in this population through analysis of mult-omic data. Integrative multiomic studies are rare in all populations, but particularly so minority populations have been overlooked. This collaborative science approach will enable us to collaborate with experts in the field which will ultimately benefit the community as well as the scientists who participate in this research. Both UNC and Vanderbilt have thriving CTSA programs.

We often hear pharma industry lament in meetings that they have great difficulty in recruiting minority populations for clinical studies. In our view, such access to these communities will require investment of thought, time, and effort in the community health infrastructure to build the needed trust and willingness to participate in these studies. Our experience suggests that pharma prefers to continue working in the large medical settings that already have the infrastructure and experience for clinical research, but where access to minority populations is limited. Changing this scenario can result in much greater opportunity to work with marginalized and minority populations. Our CCTS is proving to be a catalyst for helping to achieve this change.

It is ultimately to the benefit of all people that health research be conducted in diverse communities. This concept, effectively conducted, provides two-way education. From the community to the researcher on community priorities and concepts of their disease burden, and what can be effective approaches to research. It also provides the opportunity for the researcher to share their knowledge and insight with the community to enrich the community understanding of the importance and benefits of participating in clinical research. This two-way communication is essential for a vibrant research agenda and an atmosphere of trust and understanding of the importance of research in the community.
